# Post-coronavirus disease syndrome and disseminated microthrombosis: the role of the von Willebrand factor and antiphospholipid antibodies

**DOI:** 10.6061/clinics/2021/e2784

**Published:** 2021-03-15

**Authors:** Cristiana Sieiro-Santos, José López-Castro

**Affiliations:** I Reumatology Department, León University Hospital Complex, León, Spain.; II Internal Medicine Department, Public Hospital of Monforte, Lugo, Spain.

To the Editor,

There is sufficient evidence to confirm that severe acute respiratory syndrome coronavirus 2 (SARS-CoV-2) is a proinflammatory and prothrombogenic virus with a highly mutagenic profile. SARS-CoV-2 can produce active infection of variable duration in various organs and systems (lung, digestive system, central nervous system, skin) (1), although the pathophysiological mechanism by which disseminated microthrombosis occurs is unclear.

It has been established that the virus induces chronic oxidative stress at the endothelial level, causing the release of von Willebrand factor (vWF) multimers and generating hypercoagulability, despite the thrombopenia caused by SARS-CoV-2, which leads to an imbalance in favor of prothrombosis through increases in thrombin and D-dimer levels. In addition, the relationship between vWF levels and blood group has been known for more than a decade, with lower levels of vWF exhibited by individuals in group O and higher levels exhibited by those in group A. This is because vWF is modified by oligosaccharide chains of the antigenic determinants of the ABO system, which affects stability and activity (2). However, there is no evidence that this hypercoagulability is related to the ABO blood group or to suggest that patients with blood group A have a higher risk for thrombosis than those with blood group O, nor is there evidence that blood group A is associated with a worse prognosis for coronavirus disease (COVID-19). Nevertheless, it would be highly useful to monitor vWF as an independent prognostic marker of severe COVID-19 and risk for respiratory distress syndrome in adults (3). Hypoxic vasoocclusion and direct activation of cells by viral transduction are other mechanisms by which SARS-CoV-2 infection can lead to alterations in other coagulation parameters, such as prolonged activated partial thromboplastin time (aPTT), elevated D-dimer levels, and fibrinogen degradation products that are correlated with the severity of the disease and are associated with increased mortality (4). Many antiphospholipid antibodies (aPL) are observed in patients with COVID-19, with most studies published to date including only one aPL measurement point—generally during the acute phase—without confirmation after at least three months, as defined by the laboratory criteria for antiphospholipid syndrome (5). Lupus anticoagulant is a well-known cause of aPTT prolongation that can be detected in a significant percentage of patients with COVID-19, although it is important to be aware that aPL can appear transiently in patients with other critical and diverse illnesses/infections (*e.g.*, HIV, hepatitis C, parvovirus B19, *etc*.) because of mechanisms of molecular mimicry. The presence of these autoantibodies can lead to subclinical microthrombotic events, which explains why when patients recover from the acute clinical phase of the disease, the damaged endothelial tissue persists. Moreover, in some cases, it is reactivated in different regions of the microvasculature, such as the *vasa vasorum* of the peripheral nerves and the cerebral microvasculature, and produces a chronic proinflammatory state (endothelitis) that would condition chronic neuropathic or mnesic alterations (1). Therefore, we recommend systematic measurement of vWF and aPL in all patients hospitalized for COVID-19 to estimate their risk for unfavorable evolution.
